# Post-operative Surveillance Following Curative Resection of Colorectal Cancer in the Elderly Population in the United Kingdom: An Observational Study

**DOI:** 10.7759/cureus.49072

**Published:** 2023-11-19

**Authors:** Florence E Shekleton, William C Baker, Edward D Courtney

**Affiliations:** 1 Surgery, Royal United Hospital Bath, Bath, GBR; 2 General Surgery, Royal United Hospital Bath, Bath, GBR

**Keywords:** current guidelines, colonoscopy, frail elderly, surveillance colonoscopy, colorectal cancer

## Abstract

Background

Colorectal cancer most commonly affects the elderly population. Post-colorectal cancer surveillance aims to reduce cancer incidence and mortality, but its necessity and effectiveness are debated, especially in the elderly population. This study explores the relevance of computer tomography (CT) and colonoscopy surveillance in patients aged 75 and over who have undergone curative resection for colorectal cancer.

Methods

A retrospective analysis of prospectively collected data was conducted on patients aged 75 and over who had undergone surgical resection of colorectal cancer between November 2014 and August 2021. Data on demographics, treatment, survival, and surveillance were gathered from electronic patient records. The primary outcome was adherence to follow-up colonoscopy and CT-scan surveillance following surgery.

Results

A total of 417 patients underwent colorectal cancer surgery, with 334 included for analysis. The cohort had an average age of 81 years, with the majority receiving laparoscopic surgery and primary anastomosis. Twelve-month CT surveillance showed normal results in 281 patients (91.8%), while 24-month CT surveillance demonstrated normal findings in 244 patients (88.7%). Only 175 patients (52.4%) had colonoscopy follow-up, with 94 (53.7%) showing normal results, 74 (42.3%) demonstrating benign polyps, and two patients (1.1%) having histologically proven cancer. Reasons for not undergoing colonoscopy included declining invitations (30 patients, 19.1%) and being too frail (45 patients, 28.7%).

Conclusion

This study reinforces the notion that colonoscopy surveillance for patients over the age of 75 may have limited benefits. In an ageing population, the benefits of surveillance in terms of early detection of recurrence must be balanced against the risks of harm from the procedure, the availability of further management, cost-effectiveness, and patient preferences. An individualised approach should be adopted, potentially with colonoscopy surveillance only recommended in patients of higher risk (extramural venous invasion (EMVI)) and a low frailty score with a life expectancy over 10 years.

## Introduction

There are 56 new cases of bowel cancer per 100,000 per year in the UK population, with the highest incidence in those aged 85-89 (2016-2018) [1]. Among these, 44% of cases are female and 56% are male. Following treatment for colorectal cancer, patients are at risk of developing a local or distant recurrence or metachronous tumours and are therefore offered surveillance. Surveillance’s primary aim is to reduce colorectal cancer incidence in patients once neoplasia clearance is achieved. This is done through the identification and resection of de novo and missed polyps, preventing their progression to cancer. The secondary aim is to reduce mortality. The National Institute for Health and Care Excellence's (NICE) guidance for post-resection surveillance currently consists of carcinoembryonic antigen (CEA) blood testing, computerised tomography (CT) scans at 12 and 24 months, and colonoscopy at one and three years [2].

Surveillance, in particular colonoscopy, is associated with risks. A study of screening colonoscopies found between four and eight serious complications for every 10,000 procedures [3]. These risks include bleeding, perforation, pain, and anaesthetic reactions such as nausea and vomiting. The incidence of these complications significantly rises in elderly and frail individuals [4]. Furthermore, the associated difficulties and risks with bowel preparation disproportionately affect the elderly, including electrolyte disturbance, hypoglycaemia, and mobility issues [5, 6]. In an ageing population, the benefits of surveillance in terms of early detection of recurrence must be balanced against the risks of harm from the procedure, the availability of further management, cost-effectiveness, and patient preferences.

Recently published evidence-based guidelines for surveillance after colorectal cancer treatment concluded that there is insufficient evidence to support recommendations in patients over 75 years and that it should only be performed on an individualised basis [7]. This is reflected in the British Society of Gastroenterology (BSG)/Association of Coloproctology of Great Britain and Ireland/Public Health England post-polypectomy and post-colorectal cancer resection surveillance guidelines released in 2019, which recommended no surveillance be carried out in those over 75 years of age or with less than 10-year life expectancy [8]. Despite these guidelines, surveillance is still practised in this elderly population across the UK, including at the site of this series. In fact, the 2019 report by the BSG identified the relevance of surveillance for those over 75 years old as a key unanswered research question [8].

The aim of this study is to assess the adherence to surveillance CT scanning and, in particular, colonoscopy, following curative resection of colorectal cancer in patients over 75 years of age. Secondary analysis aims to investigate predictive factors for elderly patients with recurrence.

## Materials and methods

**Figure 1:**
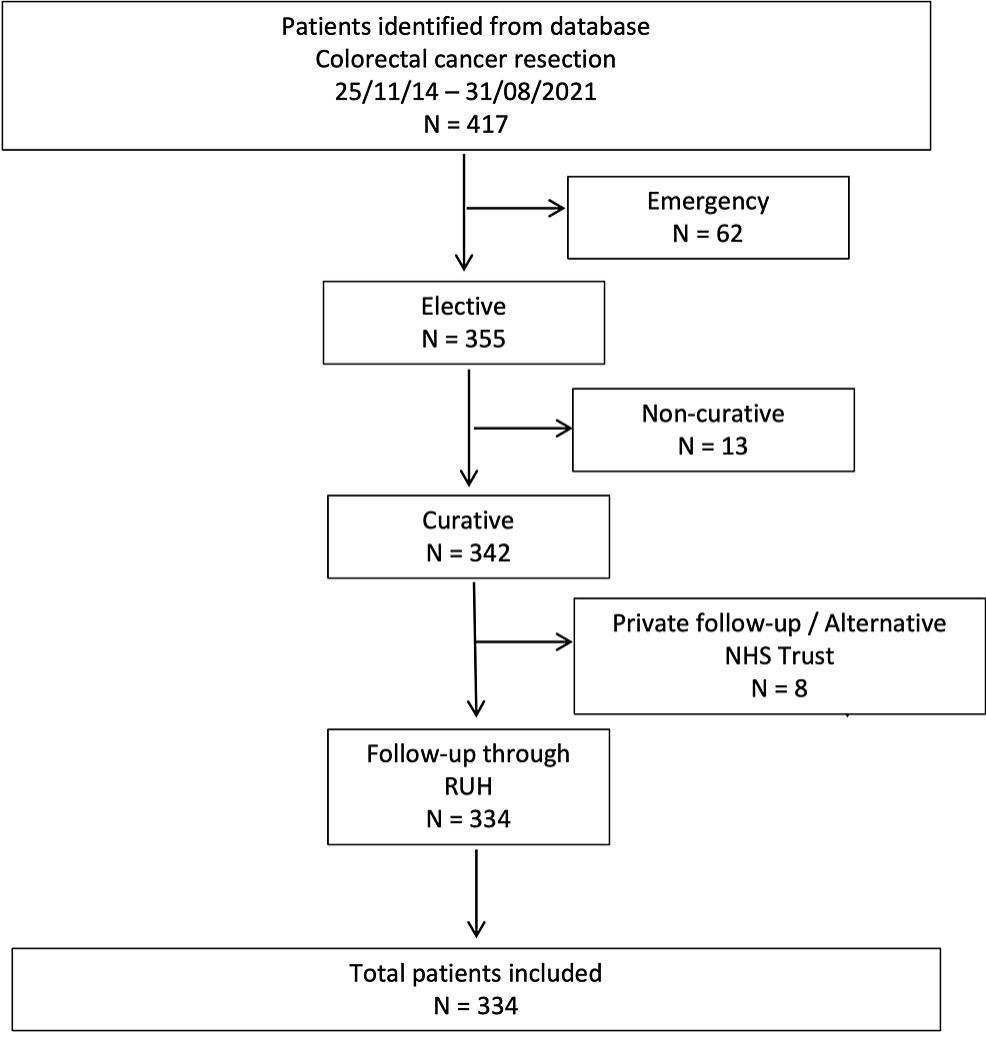
The CONSORT flow diagram demonstrates the inclusion pathway and reasons for exclusions CONSORT: Consolidated Standards of Reporting Trials; RUH: Royal United Hospital Bath

A retrospective analysis of prospectively collected data from a single district general hospital in the U.K. (Royal United Hospital, Bath, U.K.) was undertaken. All patients aged 75 years and over who had undergone surgical resection for colorectal cancer between November 2014 and August 2021 were included. Exclusion criteria were patients who had undergone emergency surgery, palliative surgery with no curative intent, and those for whom no follow-up data was available (surveillance performed at another health trust or private institution). The Consolidated Standards of Reporting Trials (CONSORT) flow diagram (Figure 1) demonstrates the inclusion pathway and reasons for exclusions.

Electronic patient records were used to collect data on age, gender, Rockwood frailty score [9], and type of operation, including laparoscopic vs. open technique, tumour location, neoadjuvant, and adjuvant therapy. The primary outcome was adherence to post-operative surveillance, which was measured as 12- and 24-monthly CT scans and a one-year colonoscopy from the date of surgery. Secondary analysis assessed the polyp size and histology if removed during surveillance colonoscopy, as well as the rate of further intervention following recurrence during surveillance. The size of the polyp was taken from the largest diameter on the histology report.

The data were extracted from electronic patient records, anonymised, and securely stored on a local database.

Following data collection, the proportions of patients who had undergone surveillance were calculated and the reason for missing data was documented. The rate of recurrence from surveillance CT scans and colonoscopy and whether further treatment was offered were analysed. Logistic regression analysis was performed to investigate whether demographic and pathological markers were predictive of recurrence from surveillance investigations on IBM SPSS Statistics software for Windows, version 28.0 (IBM Corp., Armonk, NY).

## Results

Population characteristics

Four hundred and seventeen patients underwent curative resection for colorectal cancer, of whom 334 were included in the analysis. Patient, oncological, and surgical details are displayed in Table 1.

**Table 1:** Population characteristics (n=334)

Demographic	Value	n	%
Gender	Male	170	50.9
	Female	164	49.1
Age (in years)	75-79	129	38.6
	80-84	141	42.2
	85-89	59	17.7
	90-95	5	1.5
Frailty score	1-3	290	86.8
	4-6	42	12.6
	7-9	2	0.6
Location of the tumour	Right	161	48.2
	Left	75	22.5
	Rectum	83	24.9
	Recto-sigmoid	15	4.5
Neoadjuvant therapy	Nil	304	91.0
	Chemotherapy	9	2.7
	Long-course radiotherapy (LCR)	4	1.2
	Long-course chemoradiotherapy (LCCR)	15	4.5
	Total neoadjuvant therapy (TNT)	1	0.3
Adjuvant therapy	Yes	92	27.5
	No	228	68.3
	Aborted	8	2.4
	Unknown	6	1.8
Operative approach	Laparoscopic	290	86.8
	Converted	21	6.3
	Open	22	6.6
	Laparoscopic-assisted	1	0.3
Anastomosis	Yes	270	80.8
	No	64	19.2

The majority of the cohort were male (50.9%) with a mean age of 81 years (range: 75-92 years) and a mean Rockwood frailty score of 2. Twenty-nine (8.7%) patients underwent neoadjuvant therapies, while 92 (27.5%) received adjuvant therapy. Most patients had right-sided tumours (48.2%). Operations were generally performed laparoscopically (86.8%) with a primary anastomosis (80.8%). The mean follow-up period was 66.6 months. Four patients died <90 days post-operatively (1.2%).

Computerised tomography surveillance

At the 12-monthly surveillance, 305 (91.6%) patients underwent their CT follow-up. This was unremarkable in 281 (91.8%) patients but demonstrated local recurrence in five patients (1.6%) and distant metastases in 19 patients (6.2%).

Sixty (21.5%) patients did not undergo their 24-month CT scan follow-up. For the 275 patients who did have their 24-month CT scan, no recurrent/metastatic disease was demonstrated in 88.7%, while local recurrence was seen in 1.5% and distant metastases in 5.5%. Previously known recurrence was found in 2.5% and metachronous cancer in 1.8% (Table 2).

**Table 2:** Surveillance CT findings at 12 and 24 months (n=334)

CT	12 months (n)	%	24 months (n)	%
Normal	281	91.8	244	88.7
Local recurrence	5	1.6	4	1.5
Distant recurrence	19	6.2	15	5.5
Metachronous disease	0	0.0	5	1.8
Not performed	28	9.2	59	21.5
Known recurrence	0	0.0	7	2.5

Colonoscopy surveillance

Post-operative colonoscopy surveillance was performed in 175 (52.4%) patients, and findings are demonstrated in Table 3.

**Table 3:** Surveillance colonoscopy findings (n=175)

Colonoscopy findings	n	%
Normal	94	53.7
Benign polyp	74	42.3
Polyp not retrieved	2	1.1
Colitis	3	1.7
Cancer	2	1.1

Of these, 53.7% were normal, 42.3% demonstrated polyps with benign histology, 1.1% demonstrated polyps that were not removed, 1.1% demonstrated histologically proven cancer, and 1.7% demonstrated colitis. One hundred and fifty-nine patients did not undergo colonoscopy surveillance (47.6%).

From the 42.3% of patients who had a polyp removed during colonoscopy, a total of 181 polyps were sent for histological analysis; 1.1% of polyps were not removed at the time of colonoscopy due to the patient being on anticoagulation. The mean polyp maximum diameter on the histology report was 4.4mm (range 1mm to 19mm). The majority of polyps were tubular adenomas (62.4%); 99.3% of polyps had no or low-grade dysplasia (Table 4).

**Table 4:** Histology of polyps removed at the time of colonoscopy surveillance (n=181)

Histology of polyp	n	%
Hyperplastic	44	31.2
Tubular adenoma	88	62.4
Tubulovillous	18	12.8
Sessile serrated lesion	19	13.5
Granulation tissue	1	0.7
Tubulovillous with high-grade dysplasia	1	0.7
Too small to process	1	0.7
Foreign material	2	1.4
Bowel mucosa	7	5.0

Most significantly, only 11 patients (6.2%) at their one-year colonoscopy were found to have advanced colorectal polyps as defined by the British Society of Gastroenterology (serrated polyp ³10mm, serrated polyp with dysplasia, adenoma ³10mm, adenoma with high-grade dysplasia) [8].

Reasons for not having a surveillance colonoscopy included the following: declined invitation by the patient (19.1%), patient frailty (28.7%), metastatic disease (13.4%), patient deceased (6.4%), patient unable to tolerate the procedure (1.9%), and unknown (25.5%) (Table 5).

**Table 5:** Indications for missed colonoscopy surveillance (n=160)

Reason colonoscopy not performed	n	%
Not known	40	25.5
Too frail	45	28.7
Patient declined	30	19.1
Metastatic disease	21	13.4
Died	10	6.4
Cancelled by clinician	11	7.0
Unable to tolerate	3	1.9

The gastroenterology department removed 12.2% of patients from the waiting list. In these cases, it was because their notes had been reviewed by a doctor, the surveillance was deemed no longer required, and no more specific detail was given. In some cases, colorectal surgeons then rebooked the surveillance colonoscopy.

Interventions following recurrence

Further investigation was undertaken into those patients with local or distant recurrence or metachronous tumours picked up during surveillance (Table 6).

**Table 6:** Interventions for recurrent disease detected from surveillance (n=41)

Management	n
Surgical resection	9
Palliative surgery	2
Radiotherapy	4
Hormonal therapy	2
Palliative chemotherapy	6
Curative chemotherapy	3
Nil	15

Of these, 15 opted for the best supportive care (36.6%), nine (22%) underwent surgical resection of liver, lung, or colorectal recurrence or metachronous tumours, while 4.9% had palliative surgery. Chemotherapy with curative intent was offered to 7.3%, and 14.6% had chemotherapy with palliative intent. Four (9.8%) patients had radiotherapy, and 4.9% had hormonal therapy for metachronous prostate cancer.

Prediction of recurrence

Of the 321 patients who were alive one year after surgical resection, surveillance identified 40 patients with recurrence. Binary logistic regression was used to assess if demographic (age and gender) and pathological markers from surgical resection (Tumour (T) stage (T1-T4), extramural venous invasion (EMVI), and number of positive lymph nodes) predicted recurrence (Table 7).

**Table 7:** Binary logistic regression of prediction of recurrence (n=321)

	Odds ratio (OR)	95% CI	p-value
Age	0.931	0.850 – 1.019	0.120
Gender	1.590	0.780 – 3.242	0.202
Tumour 1 (T1)	0.345	0.019 – 6.412	0.475
Tumour 2 (T2)	0.343	0.030 – 3.927	0.390
Tumour 3 (T3)	0.566	0.061 – 5.208	0.615
Tumour 4 (T4)	0.663	0.065 – 6.722	0.728
Positive lymph nodes (LNs)	1.112	0.980 – 1.262	0.100
Extramural venous invasion (EMVI)	2.608	1.121 – 6.063	0.026

The EMVI was the only variable significantly associated with local or distant recurrence during post-operative surveillance (odds ratio (OR): 2.608, 95% CI: 1.121-6.063, p=0.026).

## Discussion

This observational cohort study demonstrates that although most elderly patients over the age of 75 are proceeding with traditional CT surveillance following colorectal cancer surgery, less than half are having colonoscopy follow-up despite being recommended by the multidisciplinary team (MDT). Of those who proceeded with colonoscopy surveillance, only 1.1% of these demonstrated histologically proven cancer. Overall, this series adds weight to the growing evidence and BSG surveillance guidelines that colonoscopy surveillance in elderly patients over 75 years of age provides minimal benefit.

The debate over the optimal surveillance strategy after colorectal cancer surgery continues to persist. Jeffery et al. concluded that although an intense follow-up period offered more patients for curative surgery, there was no significant difference in overall or relapse-free survival between intense follow-up and less intense follow-up [10]. This same Cochrane review also found that intense follow-up might increase colonoscopy complications (haemorrhage and perforation) as well as being more expensive to offer. In the trust where this study was carried out (Royal United Hospital, Bath, UK, October 2023), tariffs for CT chest/abdomen/pelvis are £105, colonoscopy £530, and CEA blood test £12. Therefore, the approximate cost for complete surveillance for two years is £788 per patient. In 2021, this would have equated to a saving of £47,280.

With the rising incidence of colorectal cancer in patients under the age of 50 in Europe, there is a growing need to investigate lower gastrointestinal symptoms in those under 50 years of age. This is often with colonoscopy, which results in increased numbers of patients requiring colonoscopy in endoscopy units struggling to cope with growing demand. Therefore, reducing the need to undertake colonoscopy routinely for colorectal cancer patients aged 75 and over would allow diverting resources to more prompt diagnosis in a resource-limited healthcare system [11].

Understanding the timeline of colorectal cancer development is pivotal. The progression of polyps to cancer and the onset of clinical symptoms typically take at least a decade [12]. The risk of progression to invasive carcinoma is higher with advanced colorectal polyps. However, in this series, advanced polyps were only found in 6.2% of patients having colonoscopy one year post-resection. With an average life expectancy in the UK of 79.0 years for males and 82.9 years for females [13], surveillance colonoscopy in the over-75 age group is likely to be overtreatment for these patients, who potentially may never become symptomatic from future cancers and possibly cause harm from the complications of surveillance [14].

A large retrospective study in California showed a significantly lower incidence of colorectal cancer detected through surveillance in patients over 75 years and a significantly higher risk of post-procedure hospitalisation than younger patients [15]. These authors advocated an individualised approach to determining surveillance for elderly patients, taking into account their frailty score. This could be combined with findings from this study demonstrating that EMVI was the only variable significantly associated with local or distant recurrence during post-operative surveillance. In this study, 29% of patients with recurrence identified through surveillance did go on to have further treatment with curative intent. Clearly, there is a subgroup of over 75-year-old patients who are fit for surveillance and further treatment if required. An individualised approach, taking into account the EMVI and frailty score, could be combined as indications for screening. This offers a potential compromise between previous surveillance guidelines and the new BSG guidelines.

Colonoscopy surveillance was declined by 19.1% of our cohort of invited patients. The reason for this was not reliably documented and might range from a perceived lack of fitness for the procedure to a lack of comprehension of its necessity. Patient perspectives on colorectal cancer surveillance, as highlighted in a systematic review [16], underscore negative sentiments like anxiety, stress, unmet expectations, and a lack of support. To address these concerns, shared decision-making must take centre stage in the patient-physician dialogue, emphasising the patient's understanding of the rationale and options for surveillance. This approach could harmonise with the proposed individualised strategy, facilitating more informed decisions regarding surveillance for patients over 75.

We acknowledge that there are limitations to this series. It should be noted that a significant proportion of patients included in this study underwent surgery and follow-up during the COVID-19 pandemic. During this time, some follow-up appointments and surveillance investigations were delayed as face-to-face appointments were limited in line with restrictions, and hospital services were focused on the management of patients affected by COVID-19. However, since the timescale of this research spans the pandemic and the mean follow-up period was 66.6 months, it is thought that the effect of this is mitigated. However, with some colonoscopies being delayed by up to three years, data for the three-year colonoscopy were not included since this was unlikely to be representative with a large number of patients still on the waiting list at the time of data collection.

This was a single site for data collection. The generalisability across healthcare systems is uncertain. This research has also not taken into account whether patients were affected by hereditary colorectal syndromes, for which different guidelines and criteria for surveillance apply.

Despite the inclusion of the Rockwood frailty score, we acknowledge that other markers of frailty such as exercise tolerance (cardiopulmonary exercise testing and skeletal muscle mass) and co-morbidity classifications were not analysed. Further research is required to investigate whether these factors would influence post-operative surveillance.

## Conclusions

This study demonstrates that although most elderly patients over the age of 75 are proceeding with traditional CT surveillance following colorectal cancer surgery, less than half are having colonoscopy follow-up. Of those who proceeded with colonoscopy surveillance, only 1.1% of these demonstrated histologically proven cancer. This adds weight to the BSG surveillance guidelines that colonoscopy surveillance in elderly patients over 75 years provides minimal benefit.

Furthermore, this study found that EMVI was the only variable significantly associated with local or distant recurrence during post-operative surveillance. In an ageing population, the benefits of surveillance in terms of early detection of recurrence must be balanced against the risks of harm from the procedure, the availability of further management, cost-effectiveness, and patient preferences. An individualised approach should be adopted, potentially with colonoscopy surveillance only recommended in patients of higher risk (EMVI) and a low frailty score with a life expectancy of over 10 years.
